# Learning to detect patterns in 2 × 2 graphs

**DOI:** 10.1167/jov.26.9.6

**Published:** 2026-09-15

**Authors:** Nestor Matthews, Megan Lynn Broderick, Samantha Anne Kozlowski

**Affiliations:** 1 Department of Psychology, Denison University, Granville, OH, USA

**Keywords:** data visualization, statistics education, ensemble perception, perceptual learning, graph comprehension, factorial design, trial-and-error learning

## Abstract

Effective data visualization and statistics education both depend on helping viewers detect meaningful patterns in graphs. Here we investigated pattern detection in 2 × 2 graphs, a widely used form of data visualization. The 2 × 2 graphs display a continuous dependent variable across two predictors, each with two levels. Factor A appears on the x-axis. Factor B appears as distinct visual series, such as black versus white lines or bars. Prior research on 2 × 2 graphs examined viewers’ verbal descriptions of Factor A, Factor B, and A × B interaction patterns. Those studies revealed what people can describe but not what they can perceive. The present experiment addressed that gap by testing how viewers learn to perceptually organize Factor A, Factor B, and interaction patterns in 2 × 2 graphs. In a preregistered trial-and-error task, 416 adults learned to classify 2 × 2 line or bar graphs according to Factor A, Factor B, or A × B interaction patterns. The line and bar graphs contained identical numeric information. Two primary findings emerged. First, line graphs produced higher accuracy than bar graphs for interaction and Factor B patterns. No such advantage appeared for Factor A patterns, where accuracy remained low across graph types. Second, participants who trained on bar graphs often adopted a flawed rule. They reported Factor A and Factor B patterns only when an interaction appeared. This classification error did not occur for line graphs. Both primary findings matched preregistered predictions. These results show that data visualization choices, including graph type and predictor placement, influence the perceptual availability of patterns in 2 × 2 graphs. Clarifying those perceptual limits advances both data visualization design and statistics education.

## Introduction

Graphs translate numbers into shapes and trends. These shapes and trends support ensemble perception—the ability to extract statistical summaries from groups of similar visual elements (Alvarez & Oliva, 2008; Franconeri, 2021; Franconeri, Padilla, Shah, Zacks, & Hullman, 2021; Han, Leib, Chen, & Whitney, 2021; Kerns & Wilmer, 2021; Szafir, Haroz, Gleicher, & Franconeri, 2016). Ensemble perception enables viewers to judge the center, spread, and structure of many objects at once. Most prior work on ensemble perception examined natural scenes. Yet the same visual mechanisms also register artificial scenes, including data visualizations (Alvarez & Oliva, 2008; Franconeri, 2021; Franconeri et al., 2021; Kerns & Wilmer, 2021; Szafir et al., 2016). The present research expands that connection by linking ensemble perception to detecting mean-difference and slope-difference patterns in data visualizations.

Among data visualizations, 2 × 2 graphs hold an important role. These graphs display a continuous y-axis variable, a two-level x-axis variable (“Factor A”), and a two-level grouping variable (“Factor B”). Common examples include line graphs and bar graphs. Both rely on the Cartesian coordinate system introduced by René Descartes and popularized by William Playfair (1786, as cited in Spence, 2006). Modern software enables users to toggle between the two graph types with a single click. That informational equivalence, however, conceals psychological differences. Informationally identical line and bar graphs often produce distinct effects on viewers (Larkin & Simon, 1987).

Prior research demonstrates those differences. One set of studies (Ratwani & Trafton, 2008; Zacks & Tversky, 1999; Zhao & Gaschler, 2022) asked participants to identify numeric values from informationally identical line and bar graphs. Bar graphs yielded significantly faster reaction times than line graphs. Similarly, other studies found that, among novice graph readers, informationally identical line and bar graphs produced systematically different verbal explanations (Ali & Peebles, 2013; Shah & Freedman, 2011). Interestingly, unlike novices, graphical experts offered comparable verbal descriptions for informationally identical line and bar graphs (Peebles & Ali, 2015). That expert–novice difference underscores the need to explore how perceptual learning reshapes performance on both graph types.

The expert–novice contrast also highlights top-down influences such as familiarity with variables and general graph-reading skill. One study (Shah & Freedman, 2011) demonstrated both effects. First, students described more main effects when graphs contained familiar variables than when they contained unfamiliar ones. Second, students with stronger graphicacy generated more main-effect inferences than less-skilled peers. That task required declarative knowledge because participants described graphs verbally. Their errors, therefore, could reflect two possible sources: perception and description. The perceptual source concerns how viewers grouped the visual elements. The descriptive source concerns how viewers translated perceptions into words. Separating those two sources remains challenging. Complicating matters further, several prior declarative studies (Ali & Peebles, 2013; Peebles & Ali, 2015; Shah & Freedman, 2011) presented graphs containing labeled variable names. Some labels likely felt more familiar than others, creating semantic-familiarity bias.

Top-down factors such as semantic familiarity and translating perceptions into words influence students’ comprehension of 2 × 2 graphs. Yet graph comprehension perhaps depends even more fundamentally on how students perceptually organize a graph's elements. That perceptual organization motivates the gap our study addresses. Statistics education ultimately requires students to evaluate signal in the context of noise. For 2 × 2 graphs, the signal consists of mean differences and slope differences that correspond to Factor A, Factor B, and interaction patterns. Before students can evaluate variability, however, they must first detect those graphical patterns. The present study therefore examines a fundamental perceptual building block of graph comprehension: how viewers group elements in 2 × 2 graphs to detect Factor A, Factor B, and interaction patterns.

At the response level, this task required participants to classify graphs into one of two categories. Category learning therefore contributed to successful performance. At the same time, those classifications depended upon perceptual organization of the graph's elements. Specifically, participants needed to perceptually group elements according to proximity or similarity to extract mean differences and slope differences. The present study therefore focuses on the perceptual foundations that support those classifications.

Three distinct perceptual organizations convey information about the three patterns in any 2 × 2 graph.

The Factor A pattern occurs when a graph's left elements differ on average from its right elements. This organization follows the Gestalt perceptual grouping law of proximity (Ali & Peebles, 2013; Peebles & Ali, 2015; Shah & Freedman, 2011).

In contrast, the Gestalt perceptual grouping law of similarity organizes a graph by features that distinguish Factor B's two levels, such as black versus white. The Factor B pattern occurs when a graph's white elements differ on average from its black elements.

The interaction pattern occurs when the slopes formed by a graph's white and black elements differ.

Each organization demonstrates ensemble perception—the rapid extraction of graphical summaries such as mean differences and slope differences (Alvarez & Oliva, 2008; Franconeri, 2021; Franconeri et al., 2021; Han et al., 2021; Kerns & Wilmer, 2021; Szafir et al., 2016). Expert graph readers complete these ensemble-perception tasks efficiently. Most students struggle. Effective statistics education therefore must provide practice that strengthens perceptual organization of graphical elements. Cui and Liu (2021) review synergies among perceptual learning, ensemble perception, data visualization, and statistics education.

## The present experiment

The present experiment extends those synergies and offers new information about how viewers perceptually organize 2 × 2 graphs. Our preregistered trial-and-error task required classifying unlabeled 2 × 2 line or bar graphs using the presence or absence of Factor A, Factor B, or interaction patterns. Each participant learned one of three randomly assigned Target Factors: Factor A, Factor B, or Interaction. The sample comprised 416 online U.S. adults of diverse ages. We compared their results to preregistered predictions derived from a pilot study of college students.

## Methods

### Ethics, preregistration, and reproducibility

On March 13, 2024, Denison University's Institutional Review Board approved the experiment reported here. The experiment adheres to the October 2008 Declaration of Helsinki (World Medical Association, 2008). To minimize HARKing and P-Hacking (Kerr, 1998; Munafò et al., 2017), we preregistered the experiment's hypotheses, predictions, methods, and statistical analysis plan with the Open Science Framework on August 21, 2024, at https://osf.io/wp3hq/registrations. On August 22, 2024, we collected data with the written informed consent of each participant. To promote reproducibility, the Open Science Framework at https://osf.io/wp3hq/ contains the complete dataset and all software needed to replicate the experiment and the statistical analyses. As noted below, we distinguish preregistered analyses from post hoc analyses (Nosek, Ebersole, DeHaven, & Mellor, 2018).

### Participants

Per our preregistered method and stopping rule, the *Prolific* online crowdsourcing service recruited 420 adults residing in the United States. Among those, 416 participants finished the experiment and generated complete data files. Four other participants experienced technical malfunctions, resulting in empty data files. Our results reflect the 416 participants for whom we have complete data files. Table 1 shows the demographic information that *Prolific* provided for the 416 participants by sex, age, and ethnicity.

**Table 1.** Demographics for the 416 participants, by sex, age, and ethnicity.

Variable	Category	*n*	Percent
Sex	Female	218	52.4
	Male	196	47.1
	N.A.	2	0.5
Age	18–24	76	18.3
	25–34	152	36.5
	35–44	100	24
	45–54	64	15.4
	55–64	17	4.1
	65+	7	1.7
Ethnicity	White	244	58.7
	Black	73	17.5
	Mixed	37	8.9
	Asian	36	8.7
	Other	21	5
	NA	5	1.2

### Materials and apparatus

We initially generated Python code for the experiment using the “Builder” interface in PsychoPy 2023.2.0 (Peirce et al., 2019). The “Builder” automatically converted the PsychoPy code to PsychoJS (Version 2024.1.5) and then pushed that JavaScript to the *Pavlovia* online platform. We provided our *Prolific* participants with a web link to access the experiment's JavaScript hosted on *Pavlovia*.

In response to *Prolific's* prompt about permissible devices—“Which devices can participants use to take your study?”—we selected only the “desktop” option. Therefore, we presume that participants used desktop computers when completing the experiment online.

### 2 × 2 graph stimuli

For stimuli, we created 192 unique black-and-white 2 × 2 bar graphs and an informationally identical set of 192 black-and-white line graphs. To construct the graphs, we used the eight configurations shown in Table 2. These configurations varied according to the presence or absence of Factor A, Factor B, and interaction patterns. Configuration S1 contained no target patterns. Configurations S2–S4 each contained one target pattern. Configurations S5–S7 each contained two target patterns. Configuration S8 contained all three target patterns.

**Table 2.** The eight graph configurations and their corresponding Y/N classifications during Factor A, Factor B, and Interaction training. Participants did not receive these labels. Instead, they learned through trial-and-error feedback whether a graph belonged to category Y or category N for their assigned target factor.

Graph configuration	Factor A pattern	Factor B pattern	Interaction pattern
1	N	N	N
2	Y	N	N
3	N	Y	N
4	N	N	Y
5	Y	Y	N
6	Y	N	Y
7	N	Y	Y
8	Y	Y	Y

A Factor A pattern occurred when a graph's left and right elements differed by at least a fixed threshold. A Factor B pattern occurred when a graph's white and black elements differed by at least that threshold. An interaction pattern occurred when the slopes formed by a graph's white and black elements differed by at least that threshold. We set the threshold to 14% of the graph's y-axis range, a value obtained from our pilot study.

**Figure 1.**
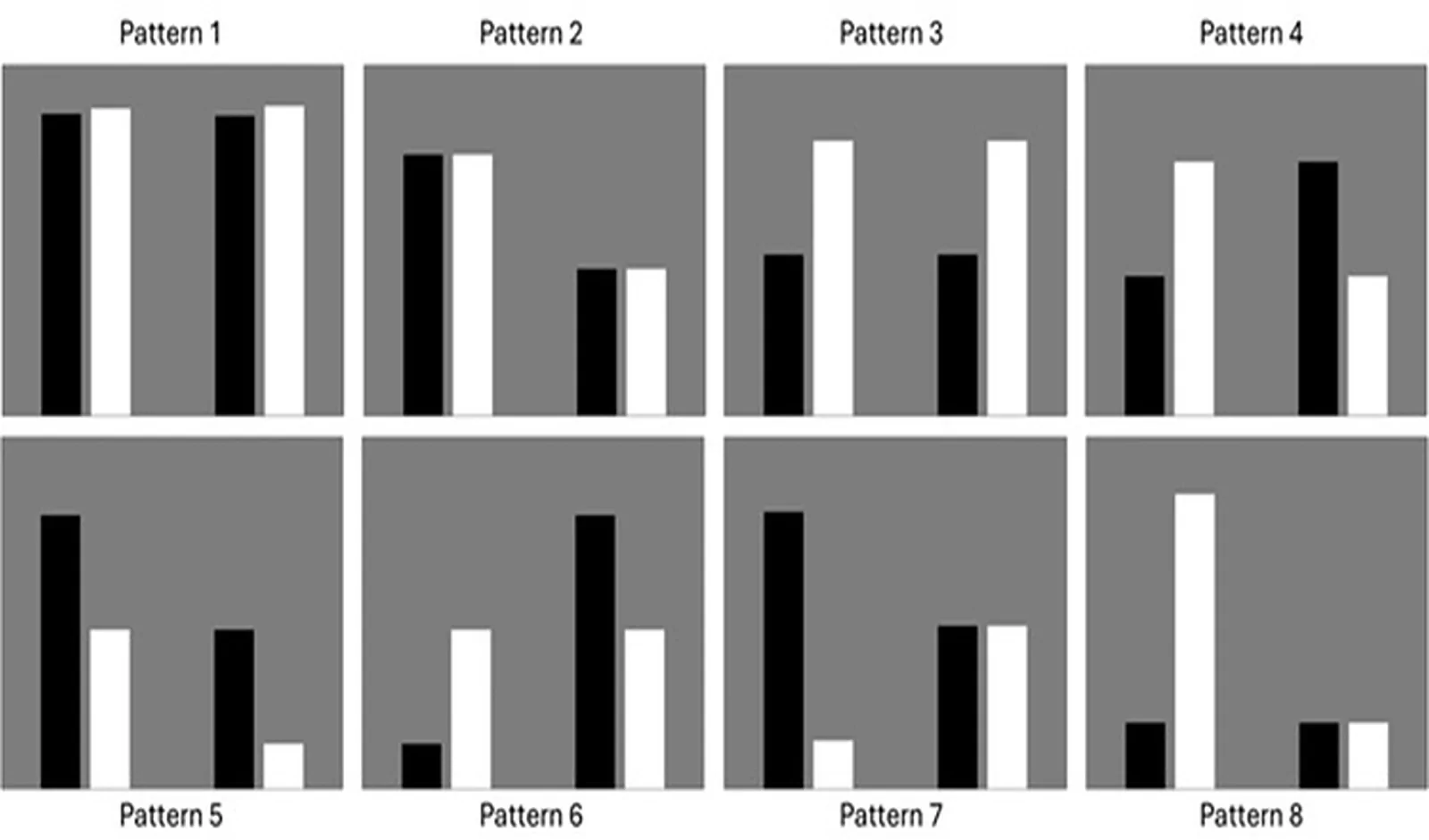
Sample bar graphs. The eight bar graphs match Table 2’s eight graph configurations and have the same numeric information as their line graph counterparts in Figure 2.

**Figure 2.**
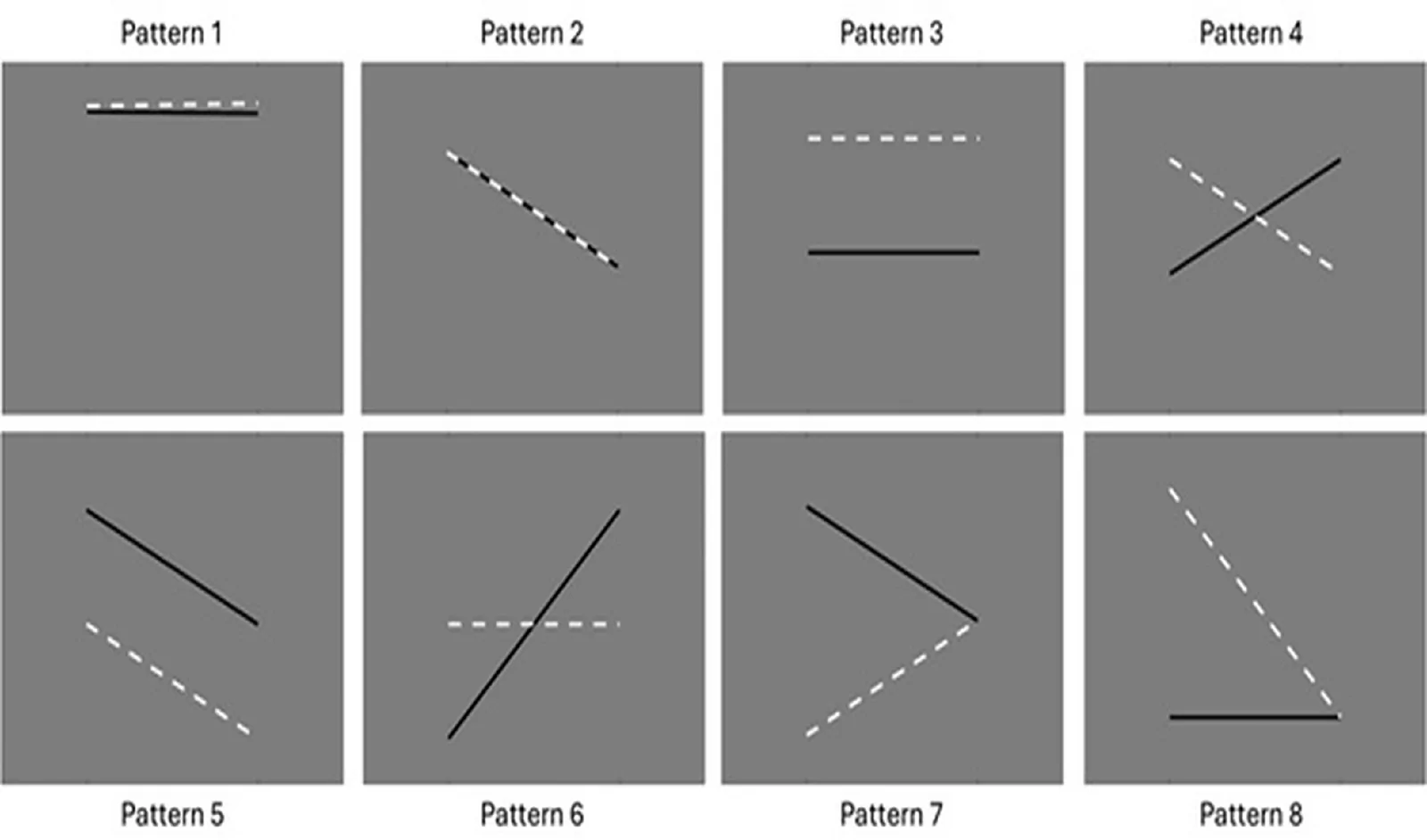
Sample line graphs. The eight line graphs match Table 2’s eight graph configurations and have the same numeric information as their bar graph counterparts in Figure 1.

Figure 1 shows sample bar graph stimuli that correspond to the eight graph configurations described in Table 2. Figure 2 shows sample line graph stimuli that contain the same numeric information as the corresponding bar graph stimuli in Figure 1.

We organized each set of 192 graphs into eight distinct 24-graph blocks. Each block formed an 8 × 3 crossing between the eight graph configurations and three magnitude levels. This crossing enhanced stimulus variety and generalizability. Specifically, Configurations S2–S8 appeared at three magnitude levels corresponding to mean differences or slope differences spanning 14%, 23.86%, or 32.41% of the y-axis range. We refer to these magnitude levels as small, medium, and large. To further enhance realistic variability, Configuration S1 contained nonzero differences that never exceeded 9.67% of the y-axis range.

During the experiment, the computer randomly shuffled the order of each block's 24 graphs for each participant. For a given target factor, correct Y classifications corresponded to graphs containing mean differences or slope differences that met or exceeded the fixed threshold. Correct N classifications corresponded to graphs containing differences that remained below that threshold.

### Task and feedback

On each trial, participants pressed either their keyboard's “N” or “Y” key to classify a 2 × 2 graph into one of two initially unknown categories. For a given target factor, category Y corresponded to graphs that contained the relevant pattern, whereas category N corresponded to graphs that lacked that pattern. Participants received one randomly assigned target factor: Factor A, Factor B, or A × B Interaction. Participants never received explicit information about their assigned target factor. Instead, they learned its classification rule through trial-and-error feedback (Table 2). Immediate feedback followed each response.

### Procedure

Participants signed up for our experiment via the *Prolific* website, which linked them to a Qualtrics-based electronic informed consent form. After giving consent, participants proceeded to our experiment on the *Pavlovia* platform.

Instructions to the participants included the following. “Each trial will begin with a simple picture. Each picture demonstrates either of two PATTERNS. If you think the image demonstrates ‘Pattern N’ press the ‘n’ key. If you think the image demonstrates ‘Pattern Y’ press the ‘y’ key. The images will demonstrate Pattern N and Pattern Y equally often. It's OK to simply guess in the beginning, when you are just learning. Prioritize ACCURACY over response speed.”

After 96 trials, participants received a rest break. They also received reminders that correct responses would occur on the “N” and “Y” keys equally often and to prioritize accuracy over speed. After completing all 192 trials, participants received U.S. $7.50 regardless of accuracy. The experiment typically required about 20 minutes to complete.

### Research design

Qualtrics randomly assigned participants across the primary independent variables of our 2 (graph type: bar, line) × 3 (target factor: A, B, interaction) between-subjects experiment. PsychoJS introduced two secondary independent variables within subjects. Those variables formed an 8 (graph configuration: see Table 2) × 3 (magnitude: small, medium, large) within-subjects design. For the dependent variable, we measured the percentage of correct responses across the 192 trials (eight 24-trial blocks).

### Stopping rule

Per our preregistration, data collection stopped after 420 participants.

### Inclusion criterion

To control for the possibility that participants responded randomly, our preregistration established an inclusion criterion. Participants met the inclusion criterion by making at least 117 correct responses across the 192 trials. This corresponded to accuracy ≥60.93% (binomial *p* < 0.001). We chose this conservative criterion to separate learners from nonlearners. The approach reduces noise from borderline performers who may not have learned the classification rule. This conservative threshold therefore understates, rather than overstates, the observed median differences between experimental conditions (graph type by target factor).

### Statistical analyses

The preregistered inclusion criterion produced substantial imbalances in the proportion of participants who met the criterion across experimental conditions. To ensure the primary analyses reflected the complete dataset, we analyzed all 416 participants via post hoc generalized linear mixed models (GLMMs). Each GLMM used a binomial error distribution and logit link function. These GLMM models accommodated the binary correct-versus-incorrect structure of the responses while avoiding Gaussian distribution assumptions. Graph Type, Target Factor, and their interactions served as fixed effects. Participants served as random intercepts. We estimated the models via maximum likelihood using the glmer() function from the lme4 package in R.

For analyses of learning across training, additional GLMMs included Block as a fixed effect. Other GLMMs examined interactions among Graph Type, Target Factor, and Graph Configuration.

For transparency, the Supplementary Materials also report the preregistered nonparametric analyses. These analyses included chi-square tests and Monte Carlo simulations that evaluated median differences and interactions among conditions. The preregistered analyses produced the same directional conclusions as the post hoc GLMM analyses reported here. The Open Science Framework contains additional computational details and all analysis code (https://osf.io/wp3hq/).

## Results

### Overall performance

**Figure 3.**
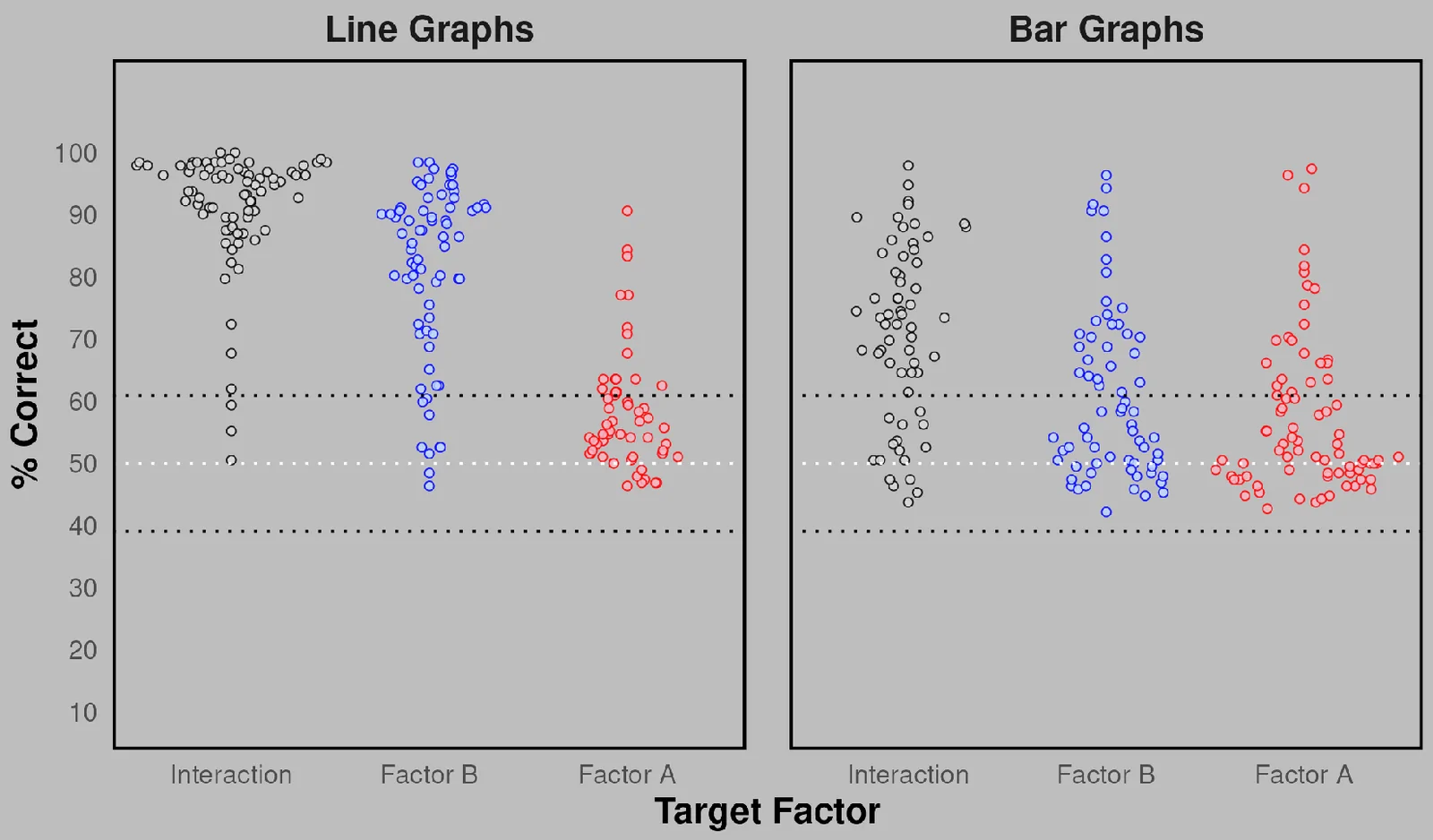
Overall task performance for all 416 participants. Each circle represents one participant's overall percent correct across 192 trials. The left panel shows line graphs. The right panel shows bar graphs. Black, blue, and red circles correspond to interaction pattern, Factor B pattern, and Factor A pattern training, respectively. The white dotted line marks chance performance (50%). The upper and lower black dotted lines mark performance significantly above or below chance (binomial *p* < 0.001).

Figure 3 visualizes performance for all 416 participants. The left panel shows participants assigned to line graphs. The right panel shows participants assigned to bar graphs. Within each panel, black, blue, and red circles correspond to participants assigned to detect interaction patterns, Factor B patterns, and Factor A patterns, respectively. The dotted white line marks chance performance (50%). The upper and lower dotted black lines mark performance significantly above or below chance (binomial *p* < 0.001).

Several features stand out immediately. First, performance depended strongly on the combination of Graph Type and Target Factor. Second, for line graphs, many participants assigned to detect interaction patterns or Factor B patterns performed well above chance. Their distributions clustered near the top of the figure. Third, participants assigned to detect Factor A patterns performed poorly with both graph types. Their distributions clustered near chance levels.

Participants assigned to detect Factor B patterns showed a contrast across graph types. Performance on Factor B patterns clustered near ceiling for line graphs but near chance for bar graphs. Consequently, many more participants exceeded the above-chance criterion with line graphs than with bar graphs.

Taken together, Figure 3 shows that successful pattern learning depended on both graph type and target factor. Some combinations supported accurate learning for most participants, whereas other combinations remained difficult despite extensive trial-and-error feedback.

### Line graph superiority effects

Our primary preregistered prediction concerned a line graph superiority effect. Specifically, we predicted line graph superiority when participants learned interaction patterns or Factor B patterns but not when they learned Factor A patterns.

**Figure 4.**
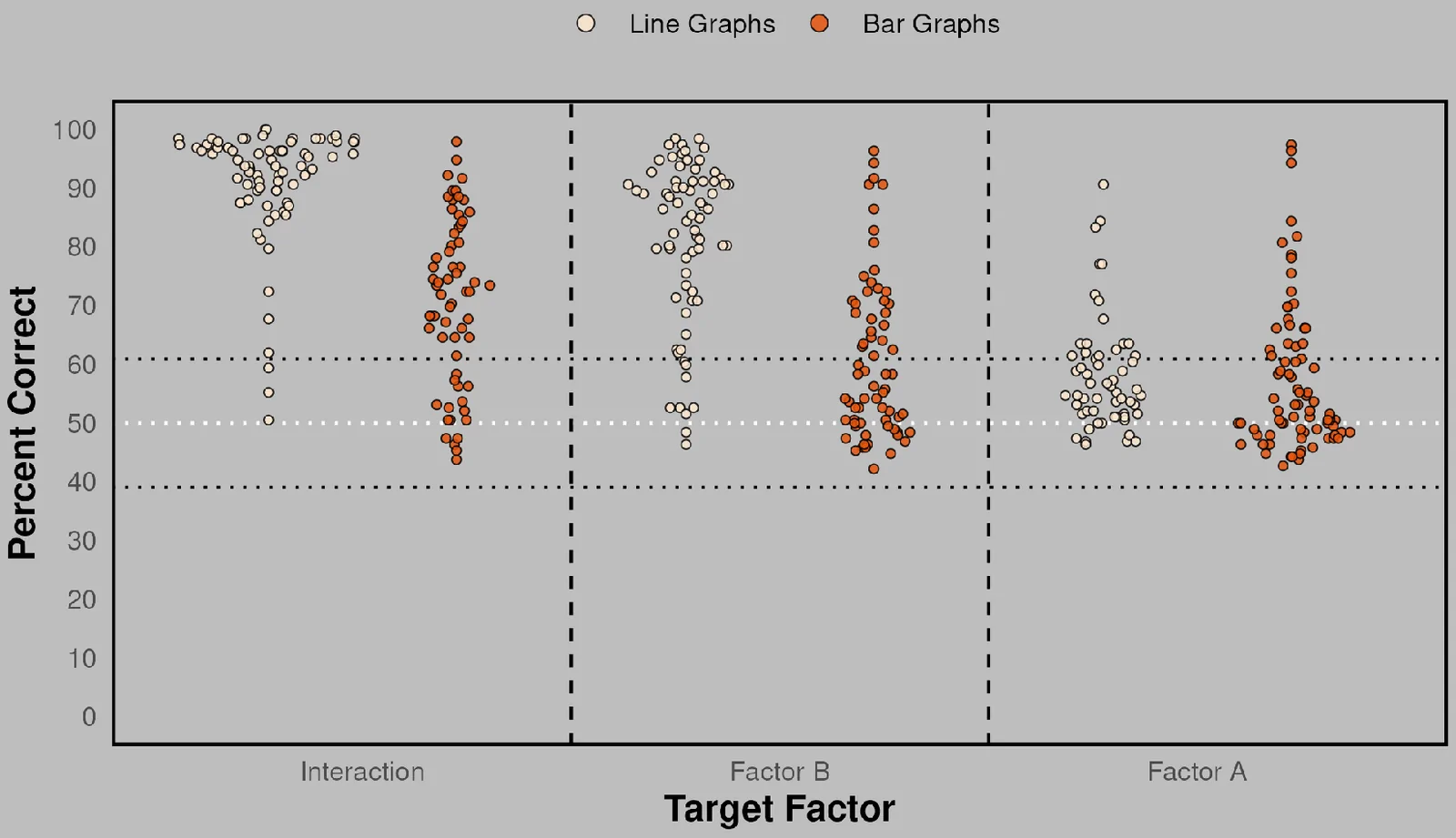
Line graph superiority effect for interactions and Factor B, not Factor A. Sina plots show percent correct for each Target Factor (Interaction, Factor B, Factor A), separately for line graphs and bar graphs. The dotted horizontal white line marks chance performance. The upper and lower dotted horizontal black lines mark performance significantly better or worse than chance. For interactions and Factor B, performance distributions shifted from negatively skewed to positively skewed when graph type changed from line graphs to bar graphs.

Figure 4 visualizes this prediction. Sina plots show overall accuracy for each Target Factor, separately for line graphs and bar graphs. Each circle represents one participant.

Several features stand out immediately. First, participants assigned to detect interaction patterns performed better with line graphs than with bar graphs. The line graph distribution clustered near ceiling, whereas the bar graph distribution shifted toward lower accuracy levels.

Second, participants assigned to detect Factor B patterns showed a similar advantage for line graphs. Again, the line graph distribution clustered near ceiling, whereas the bar graph distribution clustered much closer to chance performance.

In contrast, participants assigned to detect Factor A patterns showed little difference between graph types. Both distributions clustered near chance levels. Many participants in both conditions failed to exceed the above-chance criterion.

Taken together, Figure 4 reveals a selective line graph superiority effect. Compared to bar graphs, line graphs improved performance for interaction patterns and Factor B patterns but not for Factor A patterns.

We next evaluated these visual patterns statistically with a GLMM. The dependent variable reflected the number of correct versus incorrect responses within each 24-trial block. Fixed effects included Graph Type, Target Factor, and their interaction. Participant served as a random intercept, allowing the model to account for consistent individual differences in overall accuracy.

Planned preregistered contrasts tested whether the line graph superiority effect differed across target factors. Consistent with the preregistered prediction, the GLMM revealed a larger line graph superiority effect for interaction patterns than for Factor A patterns, β = −1.70, *z* = −8.61, *p* < 0.001. The corresponding odds ratio was 0.18, 95% CI [0.12, 0.27].

Likewise, the GLMM revealed a larger line graph superiority effect for Factor B patterns than for Factor A patterns, β = −1.20, *z* = −6.13, *p* < 0.001. The corresponding odds ratio was 0.30, 95% CI [0.21, 0.44].

Expressed differently, participants showed approximately 5.5 times greater odds of exhibiting the predicted line graph superiority effect for interaction patterns than for Factor A patterns. Participants likewise showed approximately 3.3 times greater odds of exhibiting the predicted line graph superiority effect for Factor B patterns than for Factor A patterns.

Together, these results confirm a selective line graph superiority effect. Compared to bar graphs, line graphs improved performance for interaction patterns and Factor B patterns but not for Factor A patterns.

### Learning curves

**Figure 5.**
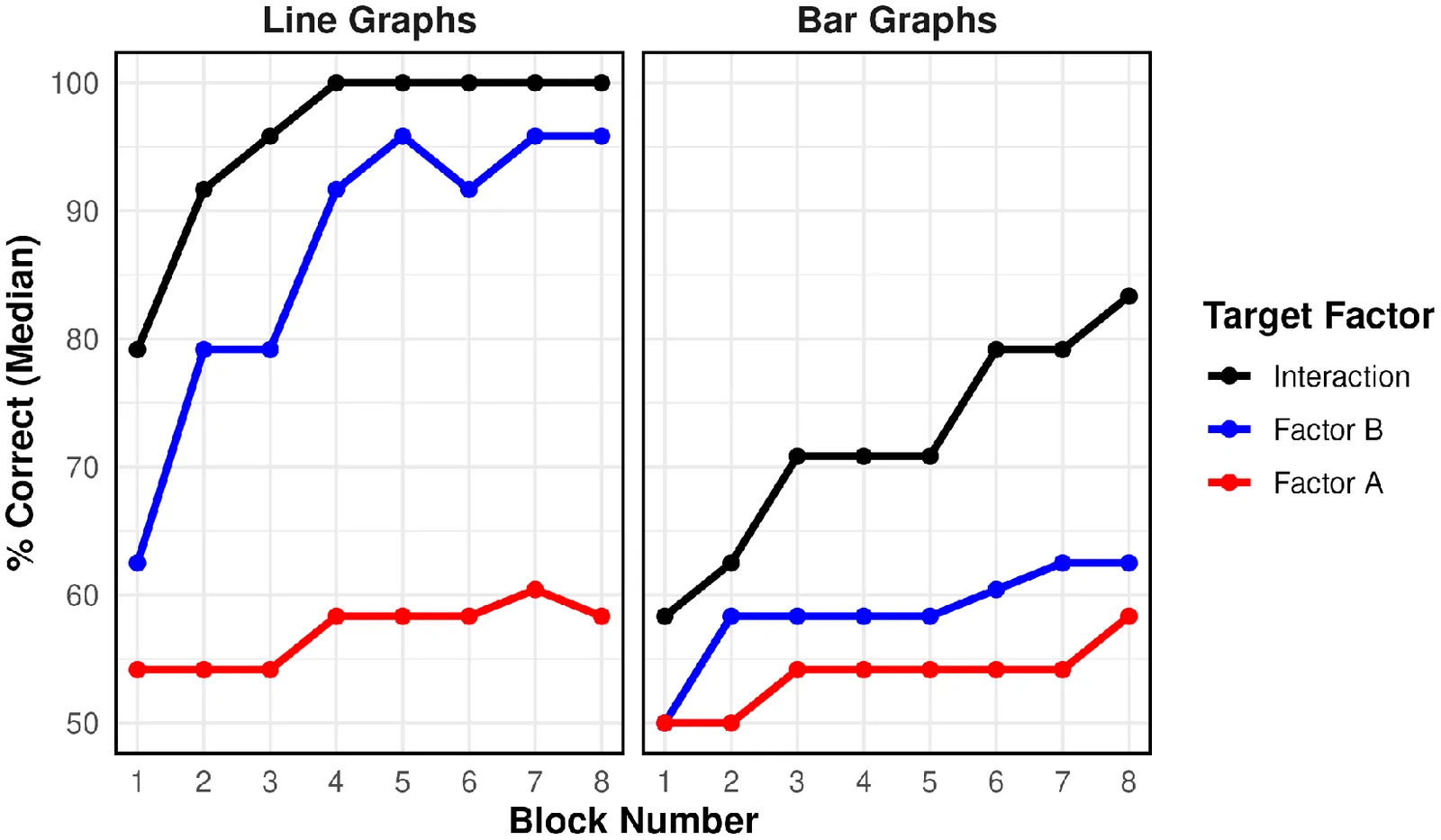
Learning curves across eight training blocks. Median percent correct appears separately for line graph training (left) and bar graph training (right). For line graphs, a stable performance hierarchy emerged across blocks: Interaction (black) > Factor B (blue) > Factor A (red). For bar graphs, performance remained lower overall, and the preregistered ordinal hierarchy did not emerge. Visual inspection reveals line graph superiority for participants assigned to detect interaction patterns and Factor B patterns. Factor A learning remained comparatively poor for both graph types.

We next examined learning across the experiment's eight training blocks. Figure 5 shows median accuracy separately for line graphs and bar graphs. Several features stand out immediately. First, participants assigned to line graphs showed substantial improvement across training blocks when learning interaction patterns and Factor B patterns. Both learning curves rose rapidly and approached ceiling performance. Second, the line graph condition produced a stable ordinal hierarchy throughout training. Participants assigned to detect interaction patterns outperformed participants assigned to detect Factor B patterns, who in turn outperformed participants assigned to detect Factor A patterns. Third, participants assigned to bar graphs showed lower performance overall. Although accuracy improved somewhat across training blocks, the learning curves remained substantially below their line graph counterparts for interaction patterns and Factor B patterns. In contrast, participants assigned to detect Factor A patterns showed relatively poor performance with both graph types. The Factor A learning curves remained near chance throughout much of training and differed little between line graphs and bar graphs.

Taken together, Figure 5 extends the earlier line graph superiority effect across the full course of learning. Line graphs facilitated learning of interaction patterns and Factor B patterns, whereas Factor A learning remained difficult regardless of graph type.

Based on the preregistered analysis plan, we first evaluated the ordinal predictions for the initial training block. The predicted ordering emerged for line graphs: Interaction > Factor B > Factor A. However, the corresponding prediction failed for bar graphs. Because the bar graph data did not satisfy the preregistered criterion for conducting the conditional Monte Carlo simulations, we did not perform those analyses.

We next evaluated Figure 5’s learning curves statistically with a GLMM. The dependent variable reflected the number of correct versus incorrect responses within each 24-trial block. Fixed effects included Graph Type, Target Factor, Block, and their interactions. Participant served as a random intercept, allowing the model to account for consistent individual differences in overall accuracy.

The GLMM revealed a significant main effect of Block, β = 0.057, *z* = 7.61, *p* < 0.001. This indicates that performance improved across training blocks overall. More importantly, the model revealed significant Graph Type × Target Factor × Block interactions for both Factor B patterns, β = 0.190, *z* = 10.62, *p* < 0.001, and interaction patterns, β = 0.205, *z* = 9.55, *p* < 0.001. These interactions indicate steeper learning trajectories for line graphs than for bar graphs when participants learned Factor B patterns or interaction patterns. In contrast, participants assigned to detect Factor A patterns showed little difference between graph types.

Targeted preregistered contrasts further confirmed the selective nature of the line graph superiority effect. Consistent with the preregistered prediction, the GLMM revealed a larger line graph superiority effect for interaction patterns than for Factor A patterns, β = −0.99, *z* = −4.96, *p* < 0.001. The corresponding odds ratio was 0.37, 95% CI [0.25, 0.55]. Likewise, the GLMM revealed a larger line graph superiority effect for Factor B patterns than for Factor A patterns, β = 0.85, *z* = 4.17, *p* < 0.001. The corresponding odds ratio was 2.35, 95% CI [1.57, 3.51].

Expressed differently, participants showed approximately 2.7 times greater odds of exhibiting a line graph superiority effect for interaction patterns than for Factor A patterns. Participants likewise showed approximately 2.4 times greater odds of exhibiting a line graph superiority effect for Factor B patterns than for Factor A patterns.

Together, the learning curve analyses converged with the earlier overall accuracy analyses. Line graphs selectively enhanced learning of interaction patterns and Factor B patterns but not Factor A patterns.

### Cognitive strategy for detecting patterns in bar graphs

The preceding analyses established a selective line graph superiority effect. We next examined the cognitive mechanism that may have produced that effect. Our preregistration predicted that many participants assigned to bar graphs would adopt an incorrect decision rule: “If there's an interaction, press Y; otherwise, press N.” This rule would generate a characteristic Pattern Number × Target Factor interaction for Patterns 2 and 6. Specifically, we predicted an upright U-shaped crossover for bar graphs but not for line graphs.

**Figure 6.**
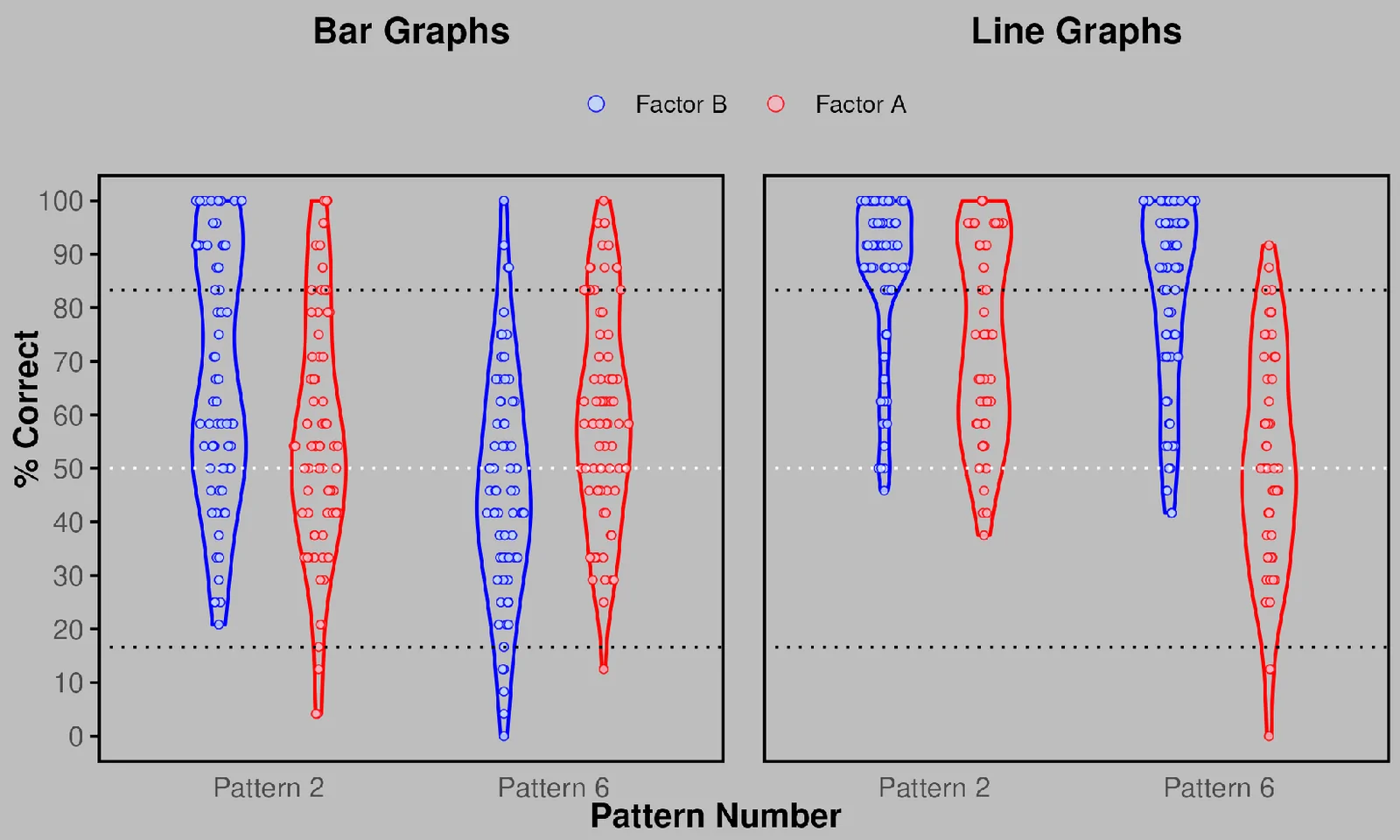
Pattern Number × Target Factor interaction for Patterns 2 and 6. Sina plots show classification accuracy for participants assigned to Factor A training (red) or Factor B training (blue). Both patterns contained large left-versus-right differences (Factor A) but no black-versus-white differences (Factor B). Pattern 6 additionally contained nonparallel slopes, whereas Pattern 2 contained only parallel slopes. This distinction produced the predicted upright U-shaped crossover for bar graphs. On Pattern 2, participants assigned to Factor B training outperformed those assigned to Factor A training. Conversely, on Pattern 6, participants assigned to Factor A training outperformed those assigned to Factor B training. This crossover largely disappeared for line graphs.

Figure 6 visualizes this prediction. The left panel shows performance on bar graph stimuli. The right panel shows performance on line graph stimuli. For bar graph stimuli, the violin centroids form the predicted upright U-shaped crossover. On Pattern 2, participants assigned to Factor B training outperformed those assigned to Factor A training. The reverse occurred on Pattern 6. This crossover matches the preregistered decision rule account.

In contrast, the upright U-shape largely disappeared for line graph stimuli. Here, the Factor B group outperformed the Factor A group on Pattern 2 and Pattern 6, substantially weakening the crossover. Simply put, the upright U-shape appeared primarily in the bar graph condition.

We next evaluated the upright U-shape statistically with a GLMM. The dependent variable reflected the number of correct versus incorrect responses for Patterns 2 and 6. Fixed effects included Graph Type, Target Factor, Pattern Number (2 vs. 6), and their interactions. Participant served as a random intercept, allowing the model to account for consistent individual differences in overall accuracy.

The critical test concerned the Graph Type × Target Factor × Pattern interaction. Consistent with the preregistered prediction, this three-way interaction reached significance, β = 1.92, *z* = 11.62, *p* < 0.001. The corresponding odds ratio was 6.85, 95% CI [4.95, 9.48]. Therefore, the Pattern Number × Target Factor interaction differed dramatically across graph types. Participants showed nearly seven times greater odds of exhibiting the predicted upright U-shape crossover for bar graphs than for line graphs. Together, these results support the proposed mechanism. When viewing bar graphs, many participants appeared to classify graphs according to the presence or absence of an interaction. That strategy produced opposite response tendencies for Patterns 2 and 6. Line graphs substantially reduced behavioral evidence for this flawed cognitive strategy.

### A conceptual replication

The previous section reported a direct replication of our pilot findings. We observed the predicted upright U-shaped interaction for bar graphs but not for line graphs. In this section, we extend that result with a conceptual replication. We replaced Patterns 2 and 6 with the structurally matched Patterns 3 and 7. This substitution replaced Factor A patterns with Factor B patterns and inverted the predicted interaction shape. Specifically, we predicted an inverted U-shaped crossover for bar graphs. All predictions in this section arose from the same decision rule as before: “If there's an interaction, press Y; otherwise, press N.”

**Figure 7.**
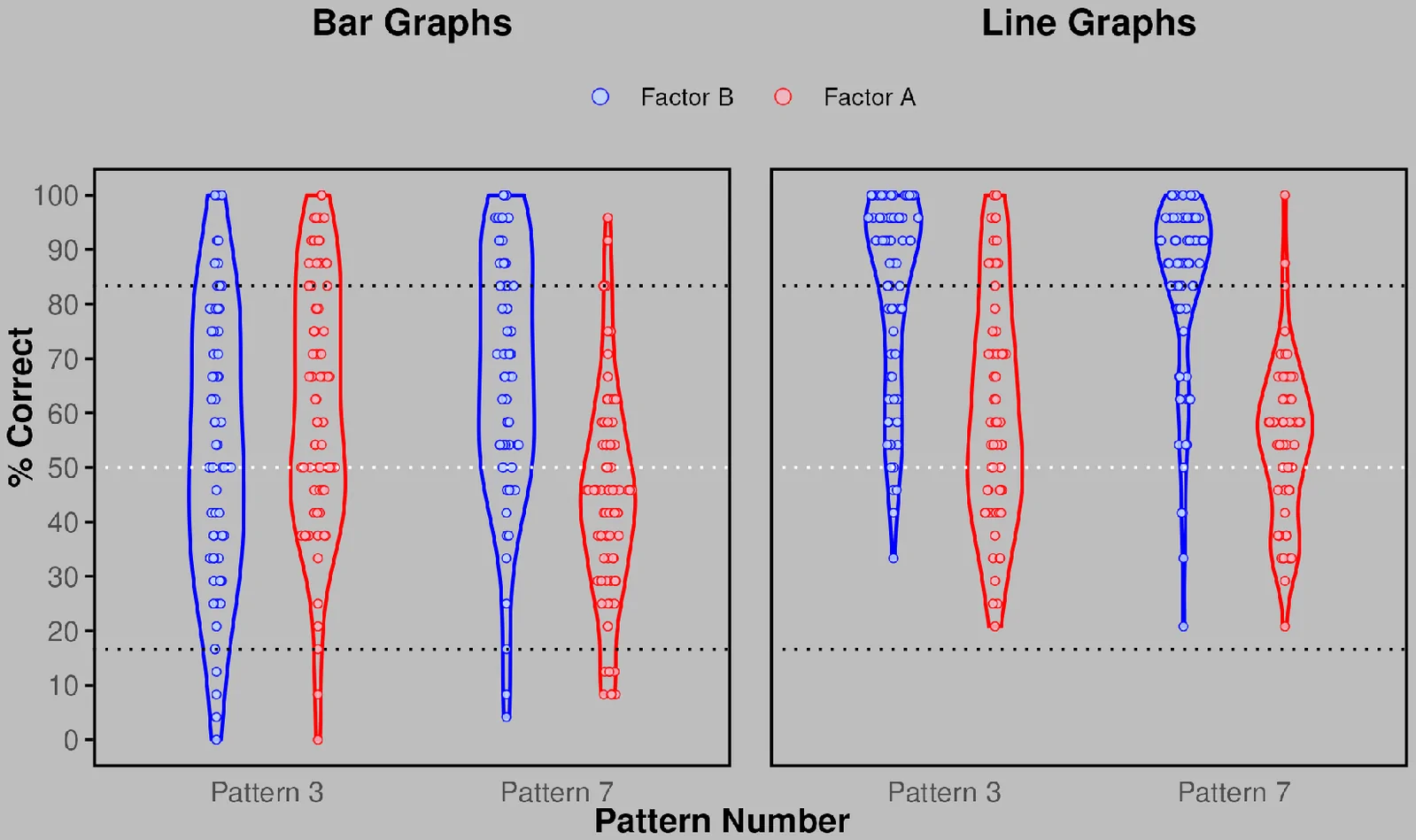
Pattern Number × Target Factor interaction for Patterns 3 and 7. Sina plots show classification accuracy for participants assigned to Factor A training (*red*) or Factor B training (*blue*). Both patterns contained large black-versus-white differences (Factor B) but no left-versus-right differences (Factor A). Pattern 7 additionally contained nonparallel slopes, whereas Pattern 3 contained only parallel slopes. This distinction produced the predicted inverted U-shaped crossover for bar graphs. On Pattern 3, participants assigned to Factor A training outperformed those assigned to Factor B training. Conversely, on Pattern 7, participants assigned to Factor B training outperformed those assigned to Factor A training. This crossover largely disappeared for line graphs.

Figure 7 visualizes this prediction. The left panel shows performance on bar graph stimuli. The right panel shows performance on line graph stimuli. For bar graph stimuli, the distributions form the predicted inverted U-shaped crossover. On Pattern 3, participants assigned to Factor A training outperformed those assigned to Factor B training. The reverse occurred on Pattern 7. This crossover matches the preregistered decision rule account.

By contrast, the inverted U-shape largely disappeared for line graph stimuli. Here, the Factor B group outperformed the Factor A group on Pattern 3 and Pattern 7, substantially weakening the crossover. Simply put, the inverted U-shape appeared primarily in the bar graph condition.

We next evaluated the specificity of the inverted U-shape statistically with a GLMM. The dependent variable reflected the number of correct versus incorrect responses for Patterns 3 and 7. Fixed effects included Graph Type, Target Factor, Pattern Number (3 vs. 7), and their interactions. Participant served as a random intercept, allowing the model to account for consistent individual differences in overall accuracy.

The critical test concerned the Graph Type × Target Factor × Pattern interaction. Consistent with the preregistered prediction, this three-way interaction reached significance, β = −1.02, *z* = −6.32, *p* < 0.001. The corresponding odds ratio was 0.36, 95% CI [0.26, 0.50]. Therefore, the Pattern Number × Target Factor interaction differed substantially across graph types. Participants showed approximately 2.8 times greater odds of exhibiting the predicted inverted U-shape crossover for bar graphs than for line graphs.

Together, these results conceptually replicated the preceding analysis. The same incorrect decision rule predicted the upright U-shape for Patterns 2 and 6 and the inverted U-shape for Patterns 3 and 7. Bar graphs again encouraged this response pattern, whereas line graphs substantially reduced behavioral evidence for this flawed cognitive strategy.

### Pattern analyses

Lastly, statistics educators may benefit from seeing how participants’ accuracy varied across the eight possible patterns that any 2 × 2 graph can assume (Table 2). Accordingly, the Supplementary Materials contain Sina plots with percent correct for every combination of Pattern Number, Graph Type, and Target Factor.

## Discussion

Modern data visualizations often use 2 × 2 line graphs or bar graphs. These graphs pervade college science courses on factorial research designs. Recognizing this, we aimed to further prior research on effective data visualization (Alvarez & Oliva, 2008; Franconeri, 2021; Franconeri et al., 2021; Kerns & Wilmer, 2021; Szafir et al., 2016) and its synergies with perceptual learning, ensemble perception, and statistics education (Cui & Liu, 2021).

Prior research provided important information about top-down influences on 2 × 2 graph comprehension. These influences include the viewer's graphicacy (Ali & Peebles, 2013; Zacks & Tversky, 1999), familiarity with the graphed variables (Ali & Peebles, 2013), and ability to translate visual input into spoken or written descriptions (Ali & Peebles, 2013; Zacks & Tversky, 1999; Zhao & Gaschler, 2022). In contrast, the present study focused on the perceptual processes that support graph comprehension, particularly ensemble perception and perceptual learning.

### Integrating ensemble perception, perceptual learning, and visual cognition

Our results extend visual cognition research by showing how ensemble perception and perceptual learning jointly support graph comprehension and statistical reasoning in 2 × 2 graphs. Statistics education ultimately requires students to evaluate signal in the context of noise. For 2 × 2 graphs, that signal consists of mean differences and slope differences that correspond to Factor A, Factor B, and interaction patterns. Before students can evaluate variability, however, they must first detect those graphical patterns. In the present experiment, participants practiced organizing line or bar graphs according to one target pattern: Factor A, Factor B, or interaction. Through trial-and-error learning, they acquired new perceptual skills for extracting mean differences and slope differences from the graphs. These findings provide new information about how adults learn categorical boundaries that distinguish meaningful graphical patterns from random variation.

At the response level, participants learned categorical boundaries for their Y/N classifications. However, those classifications depended upon the perceptual organization of the graphs’ elements. Participants assigned to detect Factor A patterns learned to organize elements according to proximity. Participants assigned to detect Factor B patterns learned to organize elements according to similarity. Participants assigned to detect interaction patterns learned to compare slopes across those visual series. We therefore view category learning and perceptual learning as complementary accounts of performance rather than competing alternatives. Category learning determined how participants mapped perceptual evidence onto responses, whereas perceptual learning determined which evidence they extracted from the graph.

Line graphs improved learning for interaction patterns and Factor B patterns. Bar graphs, in contrast, often encouraged an incorrect grouping rule that confused Factor A and Factor B patterns with interaction patterns. This distinction highlights how seemingly minor data visualization choices can reshape the perceptual groupings underlying pattern detection. In the present study, informationally identical graphs produced distinct learning trajectories, even though the category boundaries remained identical across graph types. Therefore, differences in learning reflected differences in perceptual organization rather than differences in the response categories themselves. The results align with broader evidence that visualization format alters perceptual processing during graph comprehension (Ali & Peebles, 2013; Larkin & Simon, 1987; Peebles & Ali, 2015; Ratwani & Trafton, 2008; Shah & Freedman, 2011; Zacks & Tversky, 1999; Zhao & Gaschler, 2022).

Educationally, these outcomes emphasize the value of perceptual practice alongside declarative instruction. Repeated exposure to well-structured line graphs may strengthen students’ ability to extract statistical patterns automatically. This perceptual fluency can support higher-level reasoning, bridging perceptual learning with statistical understanding. Integrating perceptual learning exercises into statistics education could therefore accelerate students’ mastery of factorial designs.

### Limitations

#### Stimulus limitation

Our 2 × 2 graphs displayed only condition means without showing within-condition variability. Future studies could add error bars or replace bar and line graphs with violin or Sina plots. Each option would visualize variability directly, revealing whether the present results generalize to graphs that display both uncertainty and central tendency.

For the present stimuli, we also intentionally omitted variable labels, units, axis values, and broader contextual information. This abstraction increased experimental control by isolating perceptual organization from semantic familiarity and domain knowledge. At the same time, real-world graphs rarely appear without contextual information. Consequently, the present findings generalize most directly to the perceptual processing of graphical structure rather than to all aspects of graph comprehension. Future studies should determine whether simple, neutral labels strengthen, weaken, or leave unchanged the line graph advantage observed here. Prior research found that familiar variables elicited different graph interpretations than unfamiliar variables (Shah & Freedman, 2011), suggesting that semantic familiarity can influence graph comprehension. Such work could also reveal whether semantic familiarity influences perceptual organization in ways that interact with graph type.

#### Ecological validity limitation

The present findings generalize most directly to novice viewers learning unfamiliar 2 × 2 graph patterns through feedback. Whether the same perceptual learning principles extend to experts, classroom instruction, or more complex visualizations remains an open question.

#### Task limitation

Our trial-and-error task probed perceptual learning without explicit instruction. Unlike declarative tasks requiring verbal descriptions, this design adapted the classic perceptual learning paradigm of Posner and Keele (1968). Participants learned an implicit grouping rule for classifying visual patterns into categories. Although effective for probing perceptual organization, this task differs from how students typically learn to interpret 2 × 2 graphs through explicit classroom instruction in statistics courses.

Participants also learned through trial-and-error feedback, a feature that differs from many real-world encounters with graphs. Feedback nevertheless resembles instructional settings in which students repeatedly practice interpreting graphical patterns and receive corrective guidance from instructors or course materials. The present task therefore may more closely align with statistics education practices than with everyday graph consumption. Future studies could compare feedback-based learning with explicit instruction to determine whether both approaches produce similar perceptual organizations.

### Future directions

#### Older adults and graph comprehension

Although *Prolific* recruited adults across a wide age range, our sample included only seven participants aged 65 or older. Replicating these procedures with a larger older-adult sample would clarify potential age differences. Many public health 2 × 2 visualizations plot age (older vs. younger) against another binary predictor, such as vaccine status (vaccinated vs. unvaccinated). Understanding how older adults perceptually organize 2 × 2 graphs could improve the accessibility of critical health information.

#### Response time

We measured accuracy but not reaction time. Future research could examine response times for correct and incorrect classifications. Such data might reveal whether detecting Factor A patterns or Factor B patterns in bar graphs requires greater effort than detecting comparable patterns in line graphs. That evidence could corroborate the nonrandom errors observed here.

#### Extended learning and expertise

The present data revealed substantial learning across training blocks. However, the line graph advantage remained evident throughout learning. Whether extended practice eliminates the line graph advantage or whether that advantage reflects a more enduring property of perceptual organization remains a question for future research. Interestingly, prior research found that, unlike graphical novices, graphical experts produced similar verbal descriptions for informationally identical line and bar graphs (Peebles & Ali, 2015). Future studies could determine whether extended perceptual learning similarly reduces graph-type differences during pattern detection and ultimately eliminates the line graph advantage.

#### Graph design and variable placement

Future studies could examine alternative graph layouts—such as violin plots or Sina plots—as well as alternative placements of variables and graphical elements. For example, researchers could vary which variable appears on the x-axis, how visual series are represented, or whether graphical elements extend vertically or horizontally. Different arrangements may encourage different perceptual organizations and learning strategies. Such work might identify graph design principles that reduce the tendency to confuse Factor A patterns, Factor B patterns, and interaction patterns.

## Conclusions

The present findings show how perceptual learning and ensemble perception shape the visual discovery of graphical relationships. Line graphs and bar graphs can carry identical numbers yet elicit different perceptual organizations. Those differences can inform data visualization choices, science education, and applied decision-making.

This research fills a longstanding gap between what viewers can describe and what they can perceive in 2 × 2 graphs. By linking ensemble perception to pattern recognition, our findings connect visual cognition with the foundations of statistical reasoning. Clarifying that perceptual bridge advances both psychological science and the effective communication of quantitative information.

## Supplementary Material

Supplement 1

Supplement 2
