# Supplementary material for: Learning to detect patterns in 2 × 2 graphs
Source: J Vis. 2026 Sep 15;26(9):6. doi: 10.1167/jov.26.9.6 (PMC13589399; doi:10.1167/jov.26.9.6)
Supplement: Supplementary file 1 [file jovi-26-9-6_s001.pdf]

S1. Accuracy for Each of the 8 Stimulus Configurations

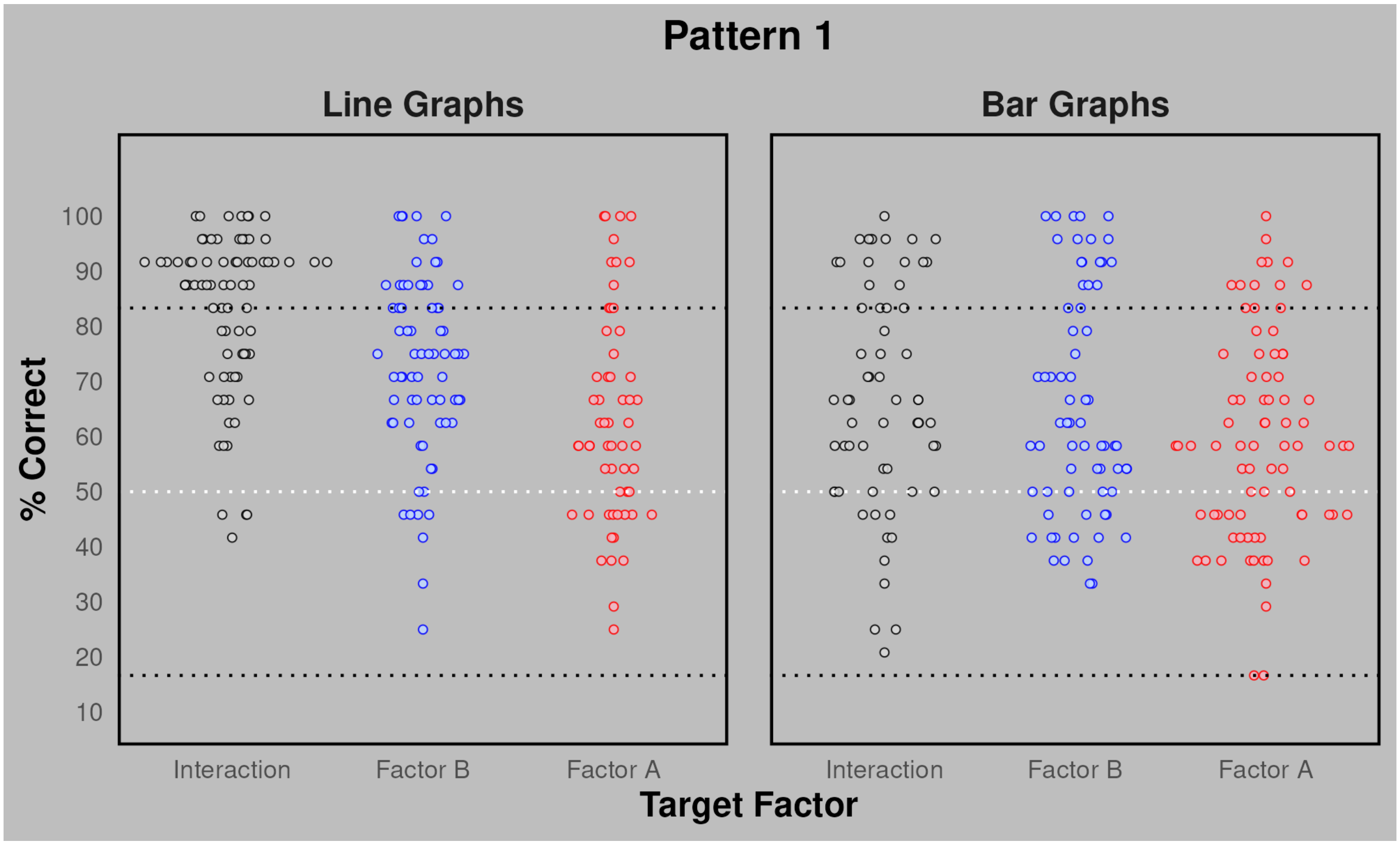

Configuration 1 Accuracy. Configuration 1 contained no target patterns.

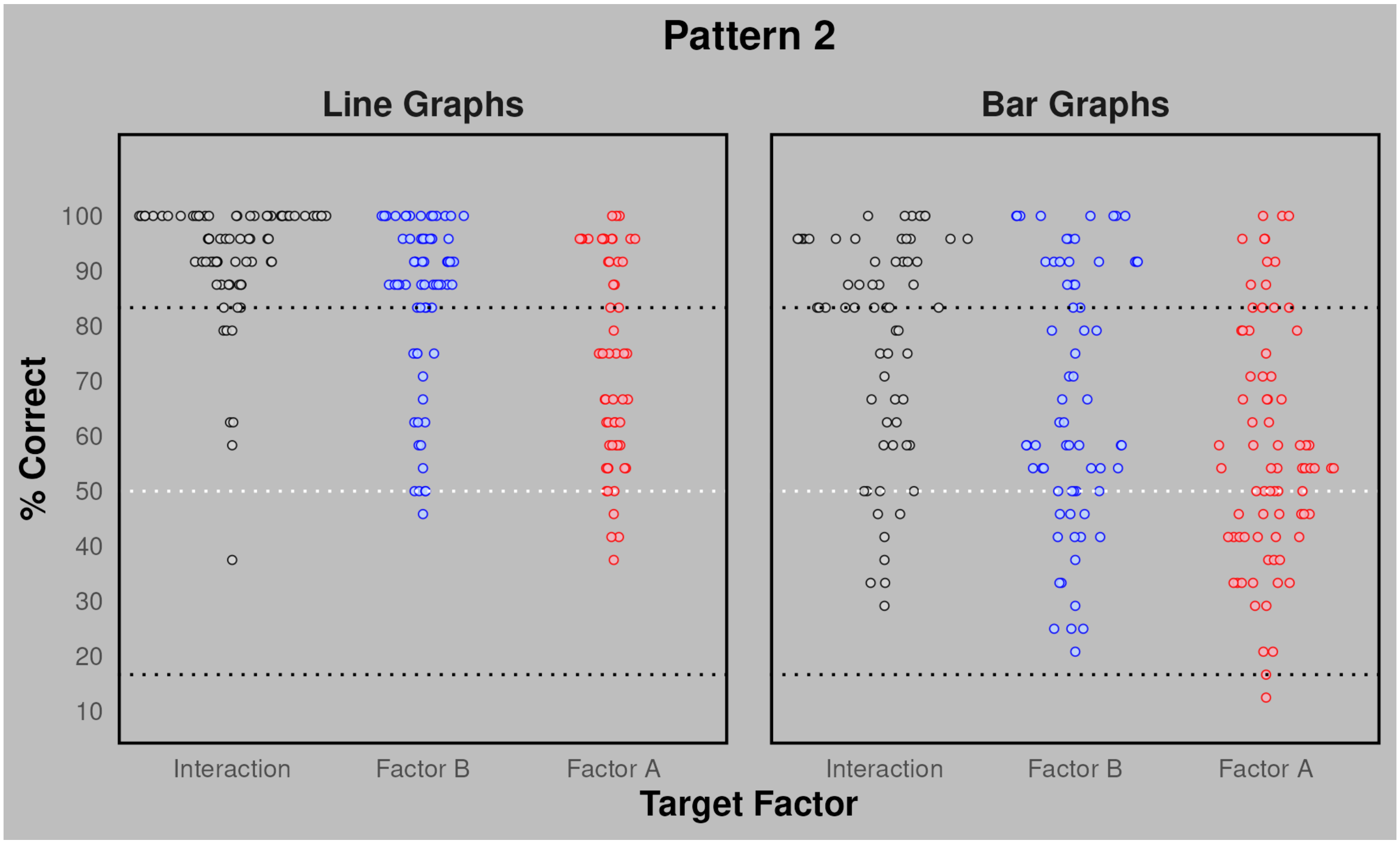

Configuration 2 Accuracy. Configuration 2 contained only a Factor A pattern.

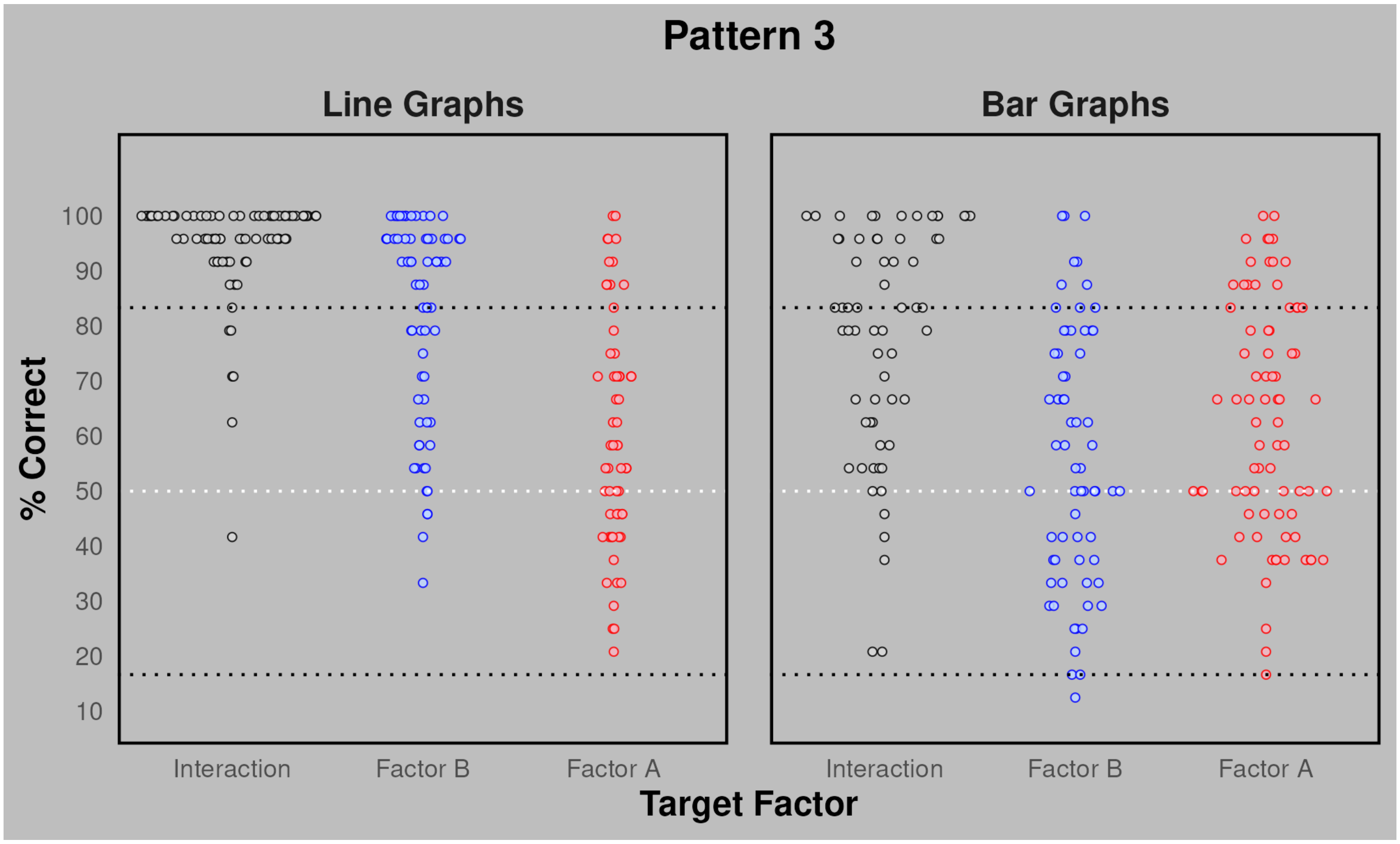

Configuration 3 Accuracy. Configuration 3 contained only a Factor B pattern.

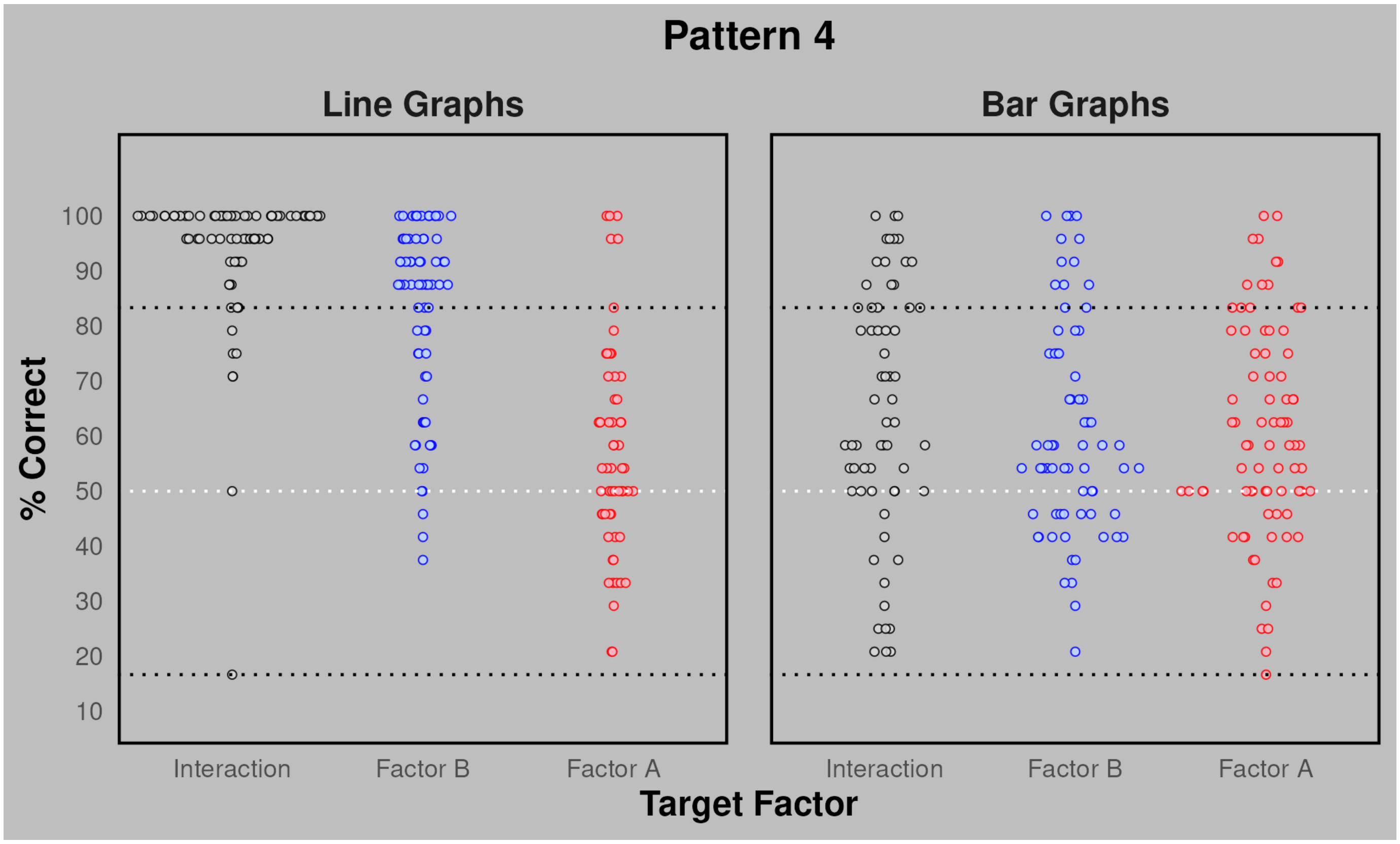

Configuration 4 Accuracy. Configuration 4 contained only an interaction pattern.

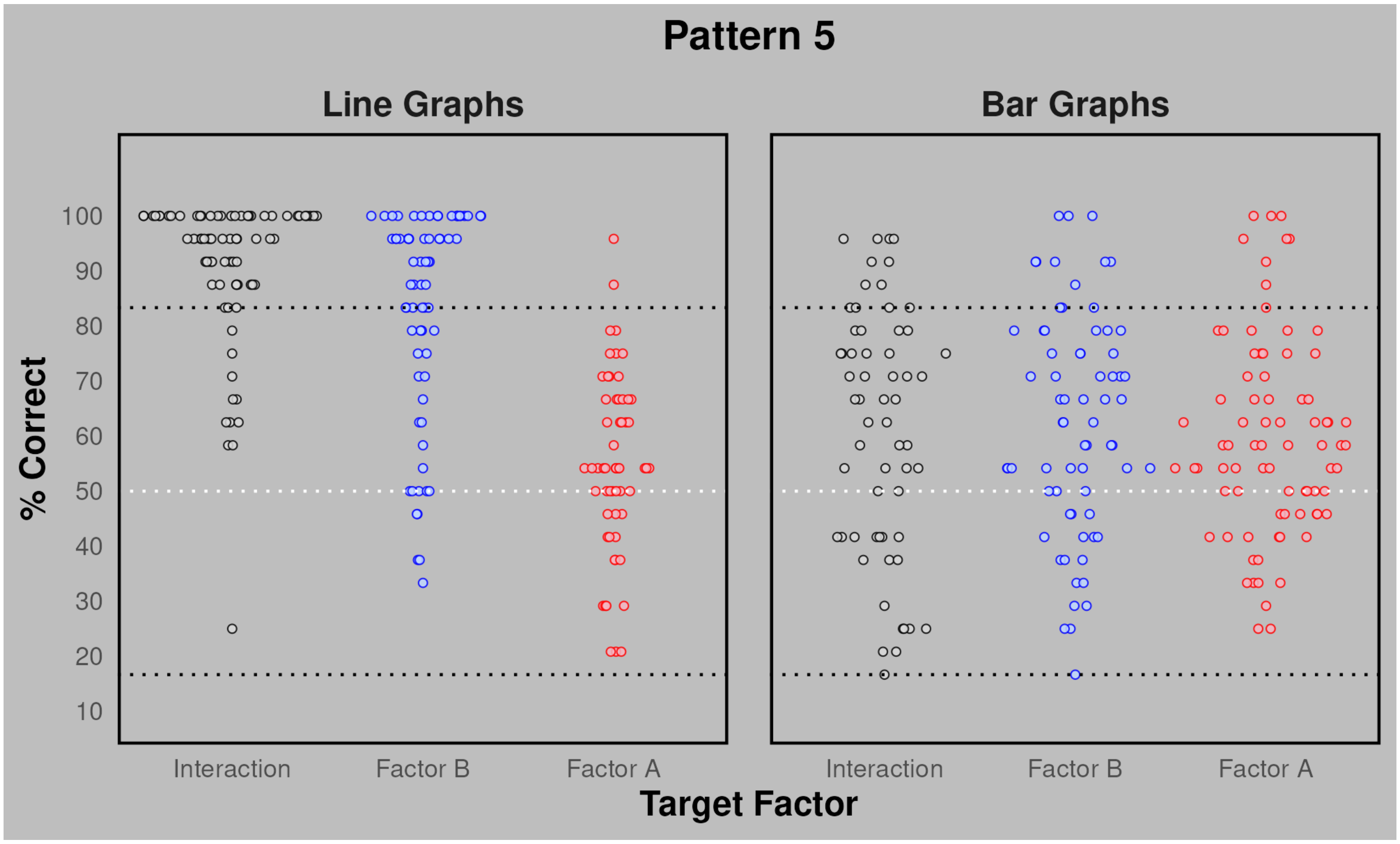

Configuration 5 Accuracy. Configuration 5 contained Factor A and Factor B patterns.

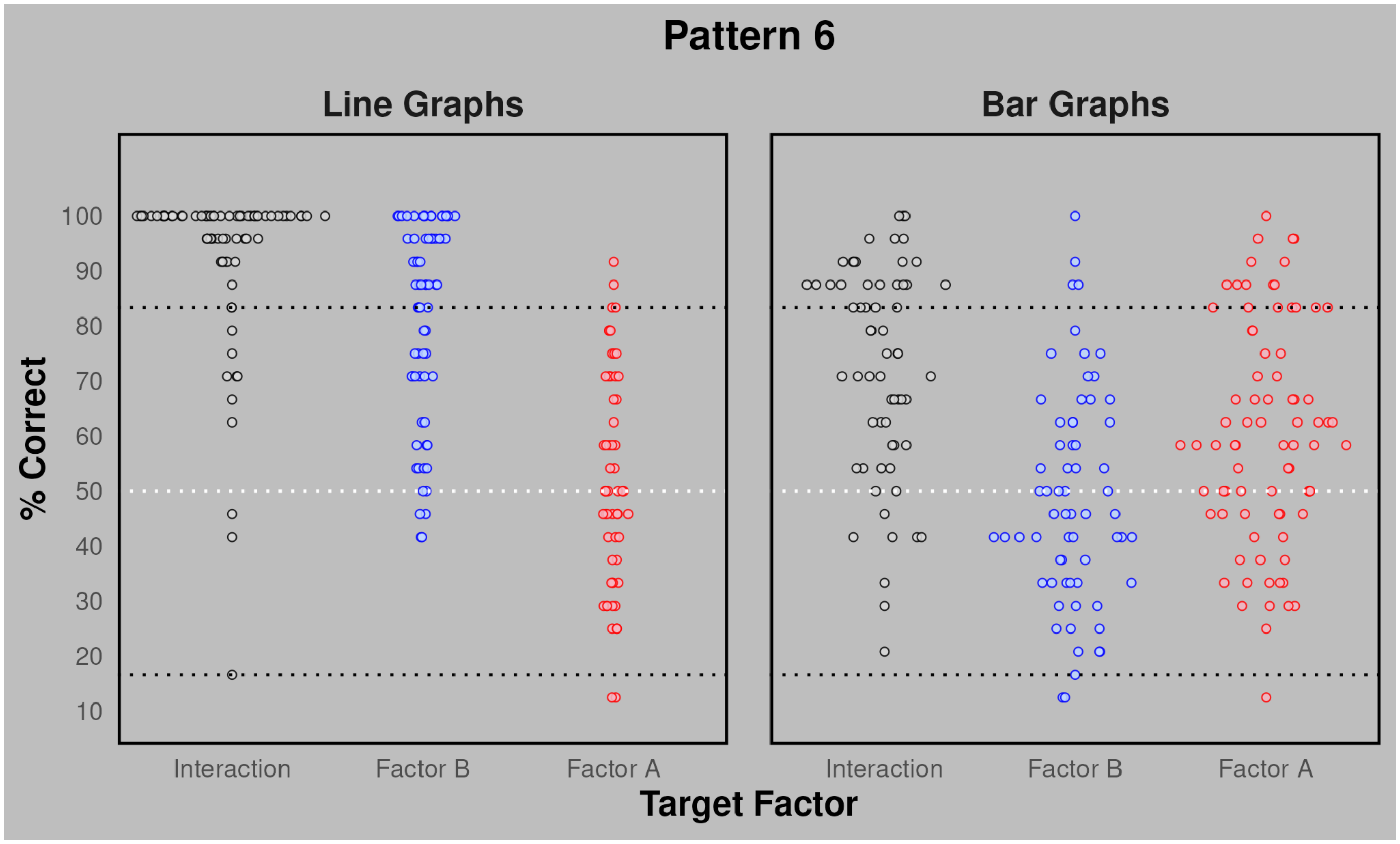

Configuration 6 Accuracy. Configuration 6 contained Factor A and interaction patterns.

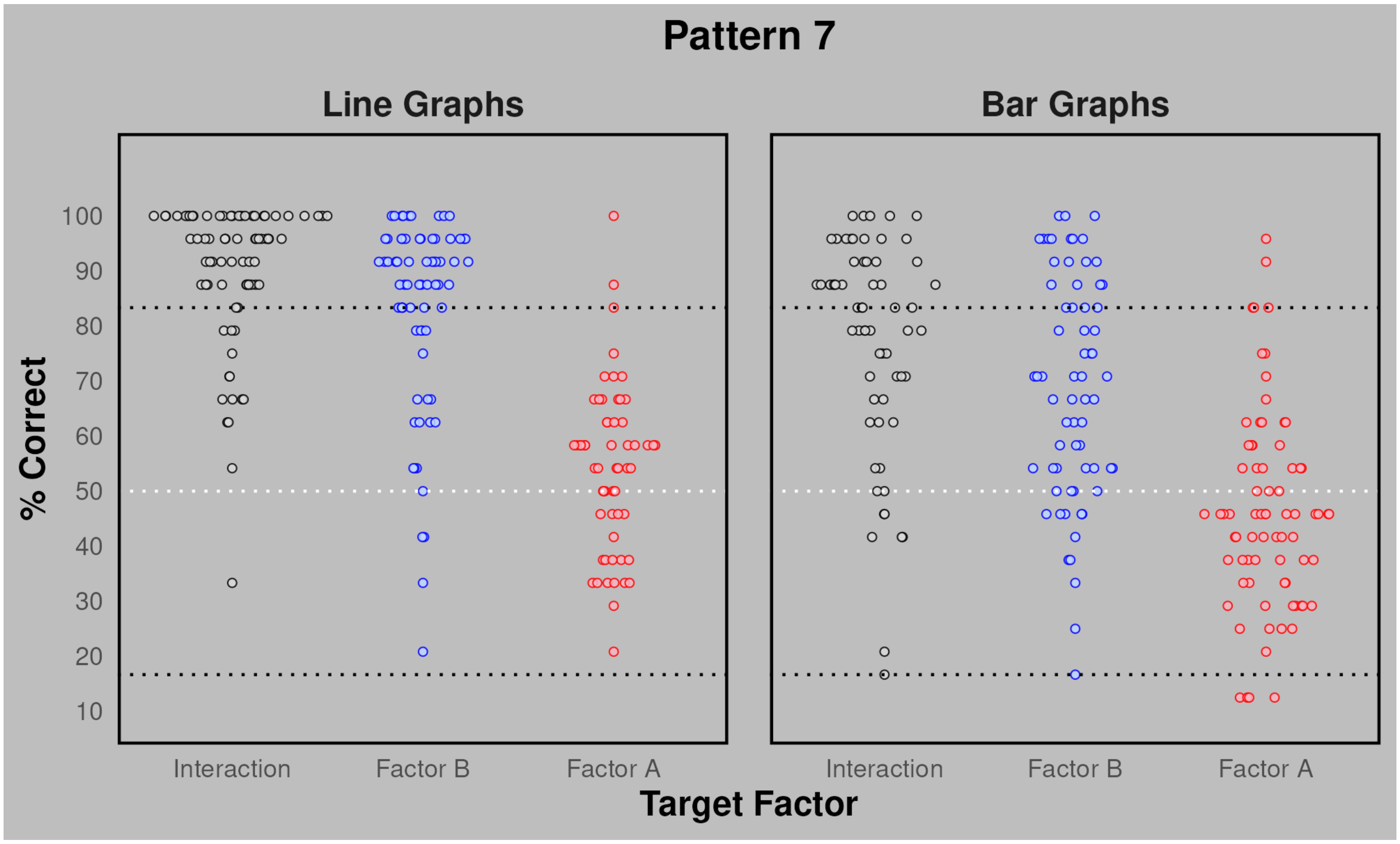

Configuration 7 Accuracy. Configuration 7 contained Factor B and interaction patterns.

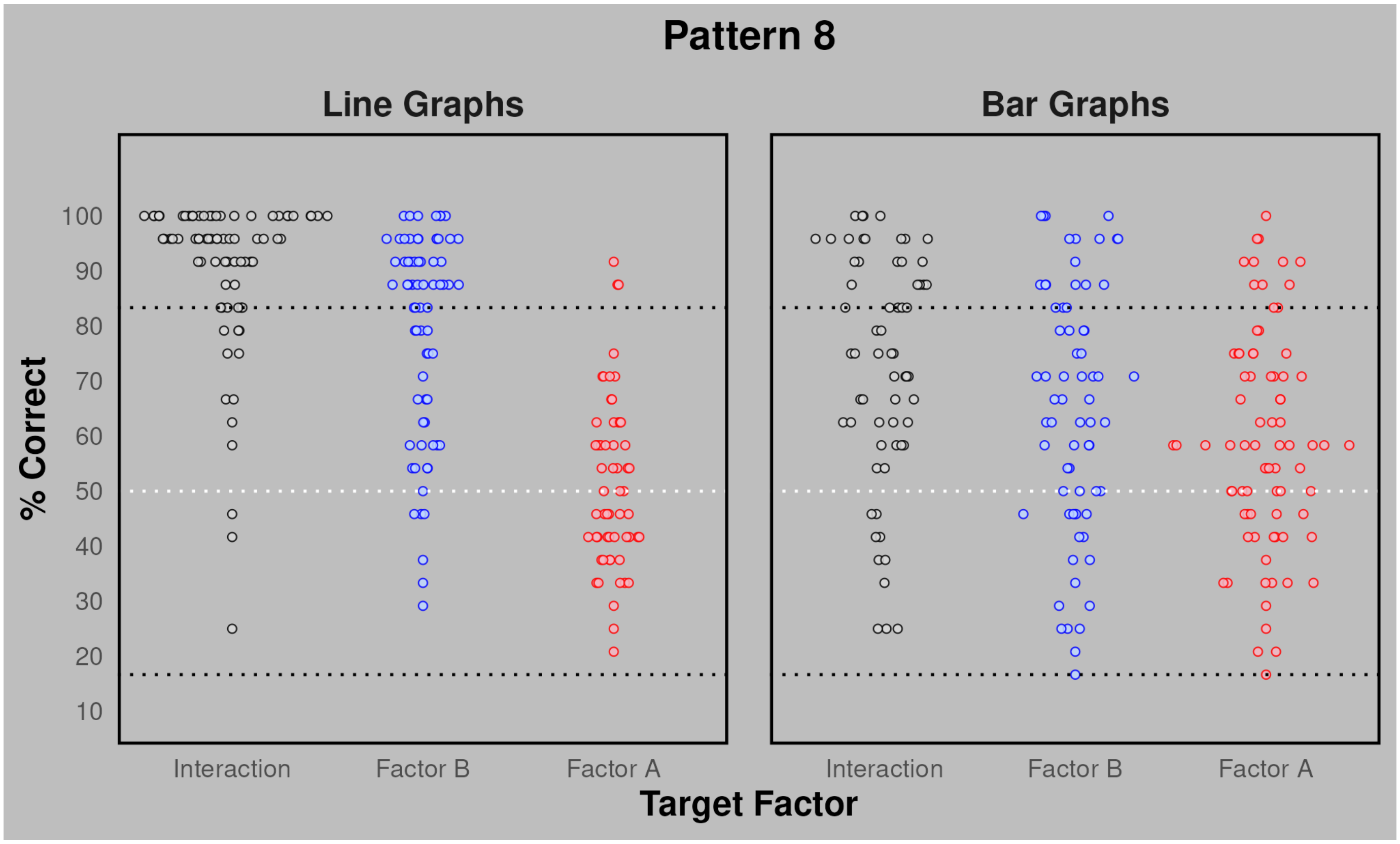

Configuration 8 Accuracy. Configuration 8 contained Factor A, Factor B, and interaction patterns.
