# Supplementary material for: Learning to detect patterns in 2 × 2 graphs
Source: J Vis. 2026 Sep 15;26(9):6. doi: 10.1167/jov.26.9.6 (PMC13589399; doi:10.1167/jov.26.9.6)
Supplement: Supplementary file 2 [file jovi-26-9-6_s002.pdf]

Preregistered Statistical Analyses

Overview

Based on skewness observed in our pilot data, we preregistered non-parametric analyses that avoided the Gaussian-distribution assumptions associated with many parametric tests. These non-parametric analyses included chi-square tests and Monte Carlo bootstrapping procedures that evaluated median differences and interactions among conditions. The Monte Carlo procedure generated a null distribution by randomly shuffling the observed data among the experimental conditions and recalculating the median difference 10,000 times. A result met the preregistered significance criterion when the observed median difference landed in the most extreme five percent of the simulated null distribution. That percentile rank also provided an intuitive effect-size metric. This procedure paralleled those described by Matthews et al. (2018) and Matthews and Folivi (2023). Additional computational details appear in the preregistration at https://osf.io/wp3hq/registrations.

Inclusion Criterion

As noted in the main text, we preregistered an inclusion criterion to reduce the influence of random responding. Participants met criterion by making at least 117 correct responses across the 192 trials. This corresponded to accuracy ≥ 60.93% correct (binomial $p < .001$). We selected this conservative threshold to distinguish learners from non-learners and reduce the influence of random responding. Consequently, the preregistered analyses likely understated, rather than overstated, median differences among experimental conditions.

Differential Attrition Across Experimental Conditions

As described in the main text, the preregistered inclusion criterion produced substantial differences in the proportion of participants meeting criterion across experimental conditions. Graph Type and Target Factor therefore influenced not only accuracy, but also the likelihood of inclusion in the preregistered analyses. This outcome raised concerns about the comparability of participants meeting criterion across conditions. Consequently, the main text reports post hoc generalized linear mixed models (GLMMs) conducted on all 416 participants.

For transparency, this Supplementary Material reports the preregistered non-parametric analyses. These analyses included chi-square tests on all participants and Monte Carlo simulations on participants who met criterion. Importantly, the preregistered analyses produced the same directional conclusions as the GLMM analyses reported in the main text.

Analyses

Graph type and target factor effects on meeting inclusion criterion

Figure 3 in the main text visualizes the proportion of participants meeting the preregistered inclusion criterion across Graph Type and Target Factor conditions. The upper dotted line marks the criterion for inclusion (≥ 60.93% correct; binomial $p < .001$).

For line graphs (Figure 3, left panel), failures to meet criterion occurred more often for Factor A than for Interaction, $X^2(1, N = 135) = 61.57$, $p < 0.001$, Cramér's $V = 0.69$. Failures to meet criterion likewise occurred more often for Factor A than for Factor B, $X^2(1, N = 129) = 40.58$, $p < 0.001$, Cramér's $V = 0.58$. These findings matched our preregistered predictions for line graphs.

For bar graphs (Figure 3, right panel), failures to meet criterion again occurred more often for Factor A than for Interaction, $X^2(1, N = 142) = 25.68$, $p < 0.001$, Cramér's V = 0.44. This outcome matched the preregistered prediction. By contrast, failures to meet criterion occurred at similar rates for Factor A and Factor B, $X^2(1, N = 147) = 1.88$, $p = 0.340$, Cramér's V = 0.13. This outcome matched the predicted direction but not the predicted magnitude.

Across conditions, the percentage of participants meeting criterion ranged from 30.4% to 87.3%. These differences indicate substantial variation in learnability across Graph Type and Target Factor combinations.

Line graph superiority effects among participants meeting criterion

Figure 4 in the main text displays line graph superiority for Interaction patterns and Factor B patterns, but not for Factor A patterns.

We preregistered predictions that line graph superiority would depend upon Target Factor. Specifically, we predicted larger line graph superiority effects for Interaction patterns and Factor B patterns than for Factor A patterns. We evaluated these predictions using preregistered Monte Carlo simulations on participants who met criterion.

First, we predicted a larger line graph superiority effect for Interaction patterns than for Factor A patterns. Consistent with this prediction, the observed median difference equaled 24.479. This value ranked at the 99.97th percentile of the simulated null distribution.

Second, we predicted a larger line graph superiority effect for Factor B patterns than for Factor A patterns. Again, the data matched the prediction. The observed median difference equaled 23.438. This value ranked at the 99.77th percentile of the simulated null distribution.

Both percentile ranks approached the upper limit of their respective null distributions. These outcomes matched the preregistered predictions and indicated large effect sizes.

Perceptual learning

Figure 5 in the main text shows the learning curves. As specified in the preregistration, we first evaluated the ordinal predictions for the initial training block. The predicted ordering emerged for line graphs: Interaction > Factor B > Factor A. However, the corresponding prediction failed for bar graphs. Because the bar graph data did not satisfy the preregistered criterion for conducting the conditional Monte Carlo simulations we did not perform those analyses.

Cognitive strategy among participants meeting criterion

Detecting Factor A patterns in bar graphs

We next examined a preregistered prediction regarding participants' strategy for detecting Factor A patterns in bar graphs. Based on pilot data, we predicted a Pattern Number × Target Factor interaction for Patterns 2 and 6. Both patterns contained large left-versus-right differences (Factor A). Pattern 6 additionally contained non-parallel slopes, whereas Pattern 2 contained only parallel slopes.

The prediction assumed that some participants would adopt a simplified rule: "If there’s an interaction, press Y; otherwise, press N." This rule predicts an upright U-shaped interaction across Patterns 2 and 6. We further predicted that this interaction would appear more strongly for bar graphs than for line graphs. Figure 6 in the main text visualizes this prediction.

We evaluated the bar-graph interaction (Figure 6’s left panel) using the preregistered Monte Carlo procedure. The observed two-way interaction value equaled +50. This value exceeded the

99.99th percentile of the simulated null distribution. The outcome matched the preregistered prediction and confirmed the upright U-shaped interaction shown in Figure 6’s left panel.

Detecting Factor A patterns in line graphs

We next applied the preregistered Monte Carlo procedure to the line graph data shown in Figure 6’s right panel. The observed two-way interaction value equaled −29.17. This value ranked at the 0th percentile of the simulated null distribution. The outcome contrasted sharply with the positively signed value observed for bar graphs and matched the preregistered prediction.

The three-way interaction: Graph Type × Pattern Number × Target Factor

We next tested the preregistered three-way interaction among Graph Type, Pattern Number, and Target Factor. The observed three-way interaction value equaled +79.17. That value ranked at the 100th percentile of the simulated null distribution. This outcome matched the preregistered prediction.

The result indicates that graph type moderated the interaction between Pattern Number and Target Factor. Although the line and bar graphs contained identical numeric information, the predicted interaction emerged much more strongly for bar graphs. Specifically, participants classifying bar graphs often appeared to rely on the presence or absence of interaction patterns. This tendency produced the upright U-shaped interaction shown in Figure 6's left panel. The corresponding line-graph data did not show this pattern (Figure 6's right panel). Together, these findings matched the preregistered prediction that graph type would influence how participants classified identical numerical data.

A conceptual replication among participants meeting criterion

Detecting Factor B patterns in bar graphs

The previous section reported a direct replication of our pilot findings. We observed the predicted three-way interaction: an upright U-shaped pattern for bar graphs (Figure 6's left panel), but not for line graphs (Figure 6's right panel). Here we extend that result with a conceptual replication.

We replaced Patterns 2 and 6 with the structurally matched Patterns 3 and 7. Patterns 2 and 6 contained large left-versus-right differences. Patterns 3 and 7 contained the corresponding black-versus-white differences. This substitution inverted the predicted interaction shape. Specifically, we predicted an inverted U-shaped interaction for bar graphs. As before, the prediction followed from the incorrect decision rule: "If there's an interaction, press Y; if not, press N."

Consistent with this prediction, Figure 7's left panel shows an inverted U-shaped interaction between Pattern Number and Target Factor. On Pattern 3, participants assigned to detect Factor B patterns performed worse than those assigned to detect Factor A patterns. The reverse occurred on Pattern 7.

The preregistered Monte Carlo analysis supported this prediction. The observed two-way interaction value equaled −81.25. That value ranked at the 0th percentile of the simulated null distribution. This outcome matched the preregistered prediction and conceptually replicated the findings for bar graphs in Figure 6.

Detecting Factor B patterns in line graphs

We next applied the preregistered Monte Carlo procedure to the line graph data shown in Figure 7’s right panel. The observed two-way interaction value equaled −29.17. This value ranked at the 0.01st percentile of the simulated null distribution.

Unlike the bar-graph data, the line-graph data did not produce an inverted U-shaped interaction. Instead, the Factor B group outperformed the Factor A group on Pattern 3 and Pattern 7. Therefore, the interaction differed substantially from the crossover pattern observed in Figure 7's left panel. This outcome matched the preregistered prediction.

The three-way interaction: Graph Type × Pattern Number × Target Factor

We next tested the preregistered three-way interaction among Graph Type, Pattern Number, and Target Factor. The observed three-way interaction value equaled −52.08. This value ranked at the 0.06th percentile of the simulated null distribution. This outcome matched the preregistered prediction.

The result indicates that Graph Type moderated the interaction between Pattern Number and Target Factor. Both graph types displayed identical numeric values. Only the visual format differed. For bar graphs (Figure 7's left panel), participants again appeared to rely on the presence or absence of interaction patterns. This tendency produced the inverted U-shaped interaction shown in Figure 7's left panel. The corresponding line-graph data produced a different pattern (Figure 7's right panel). Consequently, the observed three-way interaction matched the preregistered prediction that graph type would influence how participants classified identical numerical data.

Results summary on cognitive strategy for bar graphs

These findings support a specific and reproducible cognitive strategy for classifying patterns in 2×2 bar graphs. Based on pilot data, we preregistered the prediction that some participants would adopt the rule: "If there's an interaction, press Y; if not, press N."

This rule predicted the upright U-shaped interaction shown in Figure 6's left panel and the inverted U-shaped interaction shown in Figure 7's left panel. Both predictions matched the

observed data for bar graphs. And, as predicted, this upright to inverted U-shaped reversal did not occur for line graphs (right panels of Figures 6 and 7). Consequently, both preregistered three-way interactions matched the prediction that graph type would influence how participants classified identical numerical data.

Together, these findings replicated the pilot results both directly and conceptually. When learning to classify 2×2 bar graphs, participants appeared to prioritize interaction patterns. This strategy often impaired classification of Factor A patterns and Factor B patterns in bar graphs.

References

Matthews, N., & Folivi, F. (2023). Omit needless words: Sentence length perception. *PLOS ONE*, *18*(2), e0282146. https://doi.org/10.1371/journal.pone.0282146

Matthews, N., Welch, L., & Festa, E. (2018). Superior visual timing sensitivity in auditory but not visual world class drum corps experts. *eNeuro*, *5*(6), ENEURO.0241-18.2018. https://doi.org/10.1523/ENEURO.0241-18.2018
